# Site-Specific ^111^In-Radiolabeling of Dual-PEGylated Porous Silicon Nanoparticles and Their In Vivo Evaluation in Murine 4T1 Breast Cancer Model

**DOI:** 10.3390/pharmaceutics11120686

**Published:** 2019-12-17

**Authors:** Dave Lumen, Simo Näkki, Surachet Imlimthan, Elisavet Lambidis, Mirkka Sarparanta, Wujun Xu, Vesa-Pekka Lehto, Anu J. Airaksinen

**Affiliations:** 1 Department of Chemistry, Radiochemistry, University of Helsinki, FI-00014 Helsinki, Finland; dave.lumen@helsinki.fi (D.L.); surachet.imlimthan@helsinki.fi (S.I.); eliza.lambidis@helsinki.fi (E.L.); mirkka.sarparanta@helsinki.fi (M.S.); 2 Department of Applied Physics, University of Eastern Finland, FI-70221 Kuopio, Finland; simo.nakki@uef.fi (S.N.); vesa-pekka.lehto@uef.fi (V.-P.L.); 3 A.I. Virtanen-Institute, Department of Health Sciences, University of Eastern Finland, FI-70221 Kuopio, Finland; 4 Turku PET Centre, University of Turku, FI-20521 Turku, Finland

**Keywords:** SPECT, indium-111, click chemistry, porous silicon, IEDDA

## Abstract

Polyethylene glycol (PEG) has been successfully used for improving circulation time of several nanomaterials but prolonging the circulation of porous silicon nanoparticles (PSi NPs) has remained challenging. Here, we report a site specific radiolabeling of dual-PEGylated thermally oxidized porous silicon (DPEG-TOPSi) NPs and investigation of influence of the PEGylation on blood circulation time of TOPSi NPs. *Trans*-cyclooctene conjugated DPEG-TOPSi NPs were radiolabeled through a click reaction with [^111^In]In-DOTA-PEG_4_-tetrazine (DOTA = 1,4,7,10-tetraazacyclododecane-1,4,7,10-tetraacetic acid) and the particle behavior was evaluated in vivo in Balb/c mice bearing 4T1 murine breast cancer allografts. The dual-PEGylation significantly prolonged circulation of [^111^In]In-DPEG-TOPSi particles when compared to non-PEGylated control particles, yielding 10.8 ± 1.7% of the injected activity/g in blood at 15 min for [^111^In]In-DPEG-TOPSi NPs. The improved circulation time will be beneficial for the accumulation of targeted DPEG-TOPSi to tumors.

## 1. Introduction

Several different types of nanomaterials have been investigated as carriers for targeted delivery of therapeutic and diagnostic agents in cancer [1]. For targeted delivery, the surface of the nanocarrier is functionalized with affinity ligands such as tumor receptor binding peptides or antibody fragments [2]. For efficient recognition and retention, the carrier needs to exhibit sufficient circulation time to enable the targeting ligand to interact with the tumor cells. Unfortunately, the human body has efficient clearance mechanisms which influence the circulation time of nanoparticles (NPs) and can still make the targeted delivery with nanocarriers a challenge. The circulation time has been found to be affected by several factors, such as size, morphology, and surface chemistry of the carrier. The NPs with size beyond 100 nm are rapidly and efficiently uptaken by the liver and the spleen to so-called reticuloendothelial system (RES) [3]. Spherical and discoidal shape has been found to be preferable and engineered surface coating can further improve the residence time in circulation. The surface coating affects circulation time by delaying and preventing the opsonization process [4,5,6].

Porous silicon nanoparticles (PSi NPs) are considered as a good option for biomedical applications due to their biocompatibility and favorable physiochemical properties such as tunable particle and pore size, adjustable surface chemistry, and high degree of porosity [7,8]. The nontoxic biodegradation of PSi NPs in vivo into silicic acid and possibility to influence its biodegradation rate with surface and porosity modifications makes PSi NPs a suitable material for biological applications [9,10,11,12]. Despite of the recent successful delivery of therapeutics by multifunctional PSi NPs [13,14], there have been only a few reports for the prolongation of the NPs resident time in blood stream for intravenously administered PSi nanoparticles [15,16]. Consequently, without the improvements in the residence time in circulation, the NPs delivery to the target remains a challenge [17]. Polyethylene glycol (PEG) is widely used surface coating for improving the stealth properties of NPs, however, it has exhibited limited effect in increasing the circulation residence time of PSi nanoparticles [18]. It has been reported that the molecular weight of the PEG chain and the grafting density can enhance the stealth properties of PSi NPs [19]. A dense and thick PEG coating should improve the circulation time for example by slowing the nanoparticle phagocytosis by macrophages and by reducing non-specific protein absorption and aggregation [20]. Indeed, dualPEGylation of PSi surface by simultaneous conjugation with 0.5 kDa and 2 kDa PEG chains has been reported to prevent opsonization in vivo [15]. In cancer research, nanoparticles have demonstrated passive tumor targeting due to the enhanced permeation and retention effect (EPR) [21]. The EPR effect is a typical feature of tumors and related to their anatomical and pathophysiological differences from normal tissues [22]. Changes in tumor vasculature result in increased extravasation of nanoparticles from the blood to the tumor tissue [23]. Small size (<200 nm) as well the shape of the nanoparticle will significantly affect its potential for passive targeting [24].

Click chemistry has been widely studied in the radiopharmaceutical field because it is easily customized to different purposes [25,26]. Common for all click reactions are their fast reaction kinetics in very low concentrations and in aqueous media at room temperature which allows for use in a multitude of applications [27]. Click chemistry enables the site-specific radiolabeling of nanomaterials. Amongst all click reactions in cancer imaging and material science, the most efficient reaction reported is the [4 + 2] cycloaddition of 1,2,4,5-tetrazines and various dienophiles, known as the inverse electron-demand Diels–Alder (IEDDA) reaction, exhibiting the fast reaction kinetics, selectivity, and biocompatibility [28,29]. Each dienophile used in IEDDA has their pros and cons; for example, *trans*-cyclooctene (TCO) displays the fastest reaction kinetics but transforms relatively fast to its non-reactive *cis* form while bicyclo[6.1.0]nonyne (BCN) is more stable but the reaction kinetics are slower than compared to TCO [30,31,32].

The aim of this project was to investigate the optimal radiolabeling method for dual-PEGylated mesoporous silicon nanoparticles (DPEG-TOPSi) and to evaluate their blood circulation time and tumor accumulation in 4T1 murine breast cancer allografts. Several different labeling strategies were tested during the process, including a direct ^111^In-radiolabeling of DOTA (1,4,7,10-tetraazacyclododecane-1,4,7,10-tetraacetic acid) conjugated DPEG-TOPSi NPs and a click chemistry approach with [^111^In]In-DOTA-PEG_4_-Tz for radiolabeling of either bicyclo[6.1.0]nonyne (BCN) or *trans*-cyclooctene (TCO) conjugated DPEG-TOPSi NPs. The in vitro stability of the radiolabeled NPs was studied to ensure good radiolabel stability and for selecting the most stable radiolabeling strategy for the in vivo evaluation. The effect of PEGylation on the circulation half-life and tumor accumulation of NPs was studied with 4T1 tumor-bearing Balb/c mice with [^111^In]In-DPEG-TOPSi particles and the non-PEGylated control particles ([^111^In]In-TOPSi) by using SPECT/CT and ex vivo biodistribution studies.

## 2. Materials and Methods

### 2.1. Materials and Chemicals

All chemicals and solvents were obtained from commercial providers and they were used without further purification. *N*-(4-(1,2,4,5-tetrazin-3-yl)benzyl)-1-amino-3,6,9,12-tetraoxapentadecan-15-amide (Tz-PEG_4_-NH_2_) was purchased from Conju-Probe (San Diego, CA, U.S.A.) and 2,2′,2″-(10-(2-(2,5-dioxopyrrolidin-1-yl)-2-oxoethyl)-1,4,7,10-tetraazacyclododecane-1,4,7-triyl)triacetic acid (DOTA-NHS ester) from Macrocyclics. (1*R*,8*S*,9*S*)-Bicyclo[6.1.0]non-4-yn-9-ylmethyl *N*-succinimidyl carbonate (BCN-NHS) was purchased from Sigma-Aldrich (St. Louis, MO, U.S.A.) and *trans*-Cyclooctene-PEG_4_-NHS ester (TCO-NHS) from Jena Bioscience (Jena, Germany). Silicon wafers (p+-type, resistivity 0.007–0.02 Ωcm) were received from Okmetic Oyj (Vantaa, Finland). Dimethyl sulfoxide (DMSO), 2-propanol (IPA), fetal bovine serum (FBS), Tween**^®^** 20, triethylamine (TEA), [3-(2-Aminoethylamino)propyl]trimethoxysilane (AEPTMS), and L-glutamine were purchased from Sigma-Aldrich. Phosphate buffered saline (PBS) and penicillin/streptomycin (P/S) were obtained from HyClone (Logan, UT, U.S.A.). Dulbecco’s Modified Eagle Medium (DMEM) was obtained from Biowest (Nuaillé, France). The 2 kDa mPEG-silane and 0.5 kDa mPEG-silane were purchased from Huateng Pharma (Changsha, China) and Fluorochem (Derbyshire, UK) respectively. Trypsin was purchased from Thermo Fisher Scientific (Waltham, MA, U.S.A.). Ultrapure water (18.2 MΩ) was prepared on a Milli-Q Integral 10 water purification system. [^111^In]InCl_3_ was purchased from Mallinckrodt Medical B.V. (Petten, The Netherlands).

### 2.2. Methods

#### 2.2.1. Preparation of BCN and TCO Conjugated Nanoparticles

DPEG-TOPSi NPs were prepared as described previously from single-crystal p^+^-type Si wafers and were anodized in 1:1 (*v*/*v*) 38% hydrofluoric acid and ethanol solution with the pulse sequences [33]. Briefly, PSi NPs were oxidized at 300 °C for 2 h in ambient air to produce TOPSi and further chemically oxidized to increase the surface density of hydroxyl groups (TOPSi-OH) [34]. Mixture of 0.5-kDa and 2-kDa PEG was dissolved in anhydrous toluene, after which TOPSi-OH NPs was added to the solution [34,35]. The dispersion was heated overnight (~18 h) at 110 °C under reflux. At the end of the process, the reaction solvent was evaporated and the PEGylated PSi NPs were dispersed in ethanol. The PEGylated TOPSi (DPEG-TOPSi) NPs were rinsed with ethanol twice and 5–10 min sonication was applied during each rinsing to remove any physically adsorbed PEG. Amine groups were added to the surface of the nanoparticles (DPEG-TOPSi-NH_2_) for conjugation with the dienophiles. Briefly, DPEG-TOPSi NPs (20 mg) were dispersed in ethanol (2 mL) with AEPTMS (5 µL) and incubated for 2 h under constant rotation. Prepared DPEG-TOPSi-NH_2_ NPs were rinsed twice with ethanol and were dispersed in DMSO (5 mg/mL) and TEA (50 µL) with equal amount of BCN-NHS or TCO-NHS and incubated overnight under constant rotation. The non-PEGylated TOPSi NPs were functionalized with the same conjugation chemistry.

#### 2.2.2. Synthesis of 2,2′,2″-(10-(18-((4-(1,2,4,5-tetrazin-3-yl)phenyl)amino)-2,18-dioxo-6,9,12,15-tetraoxa-3-azaoctadecyl)-1,4,7,10-tetraazacyclododecane-1,4,7-triyl)triacetic acid (1, DOTA-PEG_4_-Tz)

Tz-PEG_4_-NH_2_ (25.4 µmol) and DOTA-NHS ester (24.0 µmol) were diluted to 2 mL dimethylformamide (DMF) and 100 µL TEA (716.0 µmol) was added to the reaction mixture and the mixture was stirred overnight at room temperature. Reaction was monitored by TLC (silica, DCM:MeOH, 10:1, *R*_f(1)_ = 0.75). The product was purified by semi-preparative HPLC (Waters Symmetry Prep™ C18, eluent: 0.1% TFA in water/ACN, ACN gradient 10% to 80%, 3 mL/min, UV detection at 270 nm, *R*_t(1)_ = 9.26 min), and evaporated to dryness, yielding a red oil. Final compound was analyzed with ^1^H-NMR (Varian Mercury 300 MHz, CD_3_OD): δ 10.33 (s, 1H), 8.57 (d, 2H), 7.60 (d, 2H), 4.54 (s, 2H), 3.92 (br, 3H), 3.80 (m, 12H), 3.64 (s, 6H), 3.60 (m, 18H), 3.35 (s, 2H) and 2.58 (t, 4H) and TOF-ESI-MS mass spectrometry (Bruker Daltonics micrOTOF). Negative ionization mode used in measurement, m/z calc. 819.4079 for C_36_H_54_N_10_O_12_^−^, found 819.3996.

#### 2.2.3. Conjugation of 1 to BCN-DPEG-TOPSi

BCN-DPEG-TOPSi particles (0.5–2 mg) were washed twice with PBS, followed by repeated centrifugations (5 min, 12,000 rpm). Particles were dispersed to PBS (pH = 7.4) and sonicated with a tip sonicator (QSonica, Newtown, CT, U.S.A) at 20% amplitude for 10 s. DOTA-PEG_4_-Tz (**1**, 0.6–1.2 mg, 0.73–1.46 µmol) in PBS (pH = 7.4, 200 µL) was added to the reaction mixture and the mixture was incubated in room temperature overnight. The conjugated particles were purified with repeated centrifugations in PBS (5 min, 12,000 rpm).

#### 2.2.4. Conjugation of 1 to TCO-DPEG-TOPSi

TCO functionalized DPEG-TOPSi particles were conjugated with DOTA-PEG_4_-Tz (1, 0.5–1.0 mg, 0.61–1.22 µmol) by incubating the particles (0.1–1.0 mg) in PBS in room temperature overnight. The conjugated particles were purified with repeated centrifugations in PBS (5 min, 12,000 rpm).

#### 2.2.5. ^111^In-radiolabeling of DPEG-TOPSi Particles

Approach A: The DOTA-PEG_4_-Tz (1) conjugated BCN or TCO functionalized particles were labeled with [^111^In]InCl_3_ (2–10 MBq) in 0.2 M NH_4_OAc buffer (100–500 µL, pH = 6.8, 37 °C) at 30 min with constant shaking followed by purification of repeated centrifugations with PBS (pH = 7.4), EtOH and PBS (5 min, 12,000 rpm). Radiochemical purity of the radiolabeled particles was controlled with i-TLC-SA (50 mmol EDTA in 0.9% NaCl) and was >98% for both particle types. Particle size for the radiolabeled particles was measured with Zetasizer Nano ZS (Malvern, Worcestershire, UK) in PBS solution.

Approach B: DOTA-PEG_4_-Tz (**1**, 0.01–0.0001 mg, 0.12–12.2 nmol) was mixed in a conical centrifuge tube with 0.2 M NH_4_OAc buffer (100–500 µL, pH = 6.8) and [^111^In]InCl_3_ (2–50 MBq). The mixture was stirred gently for 10 min at 37 °C and purified by solid phase extraction (Sep-Pak light C-18, Waters Corporation, Milford, Massachusetts, U.S.A.). The C18 cartridge was pretreated with 2 mL of EtOH and 10 mL of mQ-H_2_O. The reaction mixture was loaded to the cartridge, washed with 10 mL mQ-H_2_O and the product, [^111^In]In-DOTA-PEG_4_-Tz ([^111^In]**1**) eluted with 20% EtOH in mQ-H_2_O solution. The DPEG-TOPSi and TOPSi particles, which were functionalized either with TCO or BCN, were labeled with [^111^In]In-DOTA-PEG_4_-Tz ([^111^In]**1**) for 30 min at 37 °C with constant shaking and purified by repeated centrifugations with PBS (pH = 7.4), EtOH and PBS (5 min, 12,000 rpm). The radiochemical purity of the radiolabeled particles was determined with i-TLC-SA (50 mmol EDTA in 0.9% NaCl) and was >98% for the all particle types. Particle size for the radiolabeled particles was measured with Zetasizer Nano ZS (Malvern, Worcestershire, UK) in PBS solution.

#### 2.2.6. Particle Physicochemical Characterization

The size and ζ-potential of the nanoparticles were measured in water and the colloidal stability in PBS was determined with dynamic light scattering (Malvern, Nano ZS Zetasizer, Worcestershire, UK). Furthermore, the NP size and morphology were studied with transmission electron microscopy (TEM, Jeol JEM 2100F, Tokio, Japan) after drying a droplet of the NP suspension onto a copper grid and imaging with 200 kV beam. Chemical modifications were corroborated with transmittance Fourier-transform infrared spectroscopy (FTIR, Thermo Nicolet 8700, Cheshire, UK) and the organic content was determined with thermogravimetric analysis (TGA, TA Instruments TG Q50) by applying the heating rate of 20 °C/min up to 800 °C at N_2_ atmosphere. N_2_ sorption (Micromeritics TriStar II 3020, Norcross, GA, U.S.A.) at −196 °C was used for measuring the pore characteristics. Specific surface areas were calculated using the Brunauer–Emmet–Teller (BET) method, and Barrett–Joyner–Halenda (BJH) theory was used to obtain the pore size distributions.

#### 2.2.7. Cytotoxicity

The cytotoxicity of the nanoparticles with RAW 264.7 macrophages was evaluated. Briefly, the cells were cultured in DMEM supplemented with 10% FBS, 1% P/S and 1% L-glutamine. The cells (10,000/cm^2^) were plated on a 96-well plate and NPs (25, 50, 100 and 250 µg/mL) were incubated with the cells for 24 h. The cells were rinsed twice with PBS before applying the CellTiter-Glo assay reagent. The untreated cells and the cells incubated with 10% Tween**^®^** 20 were used as the negative and positive controls, respectively. Luminescence was measured (PerkinElmer Victor 3, Waltham, MA, U.S.A.) to determine the cell viability. The results are normalized to untreated cells (100%) and to Tween**^®^** 20 treated cells (0%).

#### 2.2.8. Radiochemical Stability of the ^111^In-Labeled Particles

The in vitro stability of the [^111^In]In-DPEG-TOPSi and [^111^In]In-TOPSi particles, prepared by both approaches (A and B), were investigated in PBS (pH = 7.4) and in human plasma (10% and 50%). For the stability tests, freshly prepared [^111^In]In-DPEG-TOPSi and [^111^In]In-TOPSi particles (0.5 mg, 1–3 MBq) were added to 1 mL of PBS or plasma solution in a protein LoBind microtube (Eppendorf) and incubated at 37 °C under constant shaking. At the predetermined time points (1 h, 2 h and 5 h) the nanoparticles were collected by centrifugation and the radioactivity of the pellet and supernatant were measured by a dose calibrator (VDC-405, Veenstra Instruments, Castel Bolognese, Italy). All assays were carried out in triplicate.

#### 2.2.9. Ex Vivo Biodistribution

All animal experiments were carried out under a project license approved by the National Board of Animal Experimentation in Finland (ESAVI/12132/04.10.07/2017, approved on 1 February 2018) and in compliance with the respective institutional, national and EU regulations and guidelines. Mice were group-housed in standard polycarbonate cages with aspen bedding and with food (Harlan) and tap water available ad libitum. Environmental conditions of a 12:12 light/dark cycle, temperature of 22 ± 1 °C, and relative humidity of 55 ± 15% were maintained throughout the study.

The ex vivo biodistribution studies were carried out in healthy (DOTA-PEG_4_-Tz, [^111^In]**1**, and [^111^In]In-TOPSi control particles,) and 4T1 tumor-bearing Balb/c female mice ([^111^In]In-DPEG-TOPSi) purchased from Charles River, aged 8–10 weeks, weighing 17–22 g (total *n* = 36). For 4T1 tumor-bearing mice, 1 × 10^6^ 4T1 cells in 50 µl of additive-free RPMI-1640 medium were inoculated to the right inguinal mammary fat pad under 2.5% isoflurane anesthesia in medical air:oxygen carrier (3:2, 1 L/min). In addition, local anesthesia infiltrated at the incision site (100 µL of 1:1 mixture of 5 mg/mL bupivacaine and 10 mg/mL lidocaine). Mice received carprofen 5 mg/kg (Norocarp, 50 mg/mL, Norbrook Laboratories Ltd**.**) subcutaneously before the surgery and at 24 h and 48 h after the surgery. Tumors were allowed to growth for 8–11 days before the administration of the radiolabeled NPs. [^111^In]In-DPEG-TOPSi (100 µg) in 200 µL PBS (pH = 7.4) and the control particles, [^111^In]In-TOPSi (100 µg) in 200 µL PBS-5% Solutol HS 15, were administered intravenously to the tail vein. The mice were sacrificed at the predetermined time points by CO_2_ asphyxiation followed by cervical dislocation. Tissues were collected at 5 min, 1 h, 4 h, 24 h, and 48 h time points for [^111^In]In-DPEG-TOPSi particles and for [^111^In]In-TOPSi particles at 30 min and 1 h time points (*n* = 4 at each time point). The circulation half-life of the particles was determined from venous blood samples by collecting a droplet from the tail vein with a needle at 5 min, 15 min, and 30 min after administration for both particles. The collected tissue samples were weighted individually, and their radioactivity measured by using a gamma counter (Perkin Elmer Life Sciences, Waltham, MA, U.S.A.).

#### 2.2.10. In Vivo SPECT/CT Imaging

4T1 tumor-bearing mice (*n* = 4) were intravenously injected with [^111^In]In-DPEG-TOPSi (2–4 MBq, 100 µg in 200 µL PBS, pH = 7.4) and scanned with a dedicated small animal SPECT/CT scanner (Bioscan NanoSPECT/CT, Mediso, Budapest, Hungary) at 1 h (dynamic scan), 5 h (static scan), and 24 h (static scan) after the administration of the radiolabeled NPs. The SPECT/CT scans were carried out under 1.5–2.5% isoflurane anesthesia in oxygen carrier. The images were analyzed with VivoQuant program (InviCRO LLC, Arlington, VA, U.S.A).

#### 2.2.11. Autoradiography

Tumors harvested at the 1 h, 4 h, and 24 h time points after the intravenous injection of [^111^In]In-DPEG-TOPSi particles were snap frozen in dry ice cold isopentane and embedded in tissue freezing medium before sectioned with a cryostat microtome (Leica CM1950, Leica Biosystems, Wetzlar, Germany) to 10 µm thick sections and collected to a glass slide (SuperFrost Plus, VWR). The sections were scanned with a real-time digital autoradiography ai4r BeaQuant system (Nantes, France) for 24 h [36]. Same tumor sections were subsequently stained with hematoxylin–eosin (H&E) and scanned at the Finnish Centre for Laboratory Animal Pathology (FCLAP).

## 3. Results and Discussion

### 3.1. Preparation of the DPEG-TOPSi Nanoparticles

**Figure 1:**
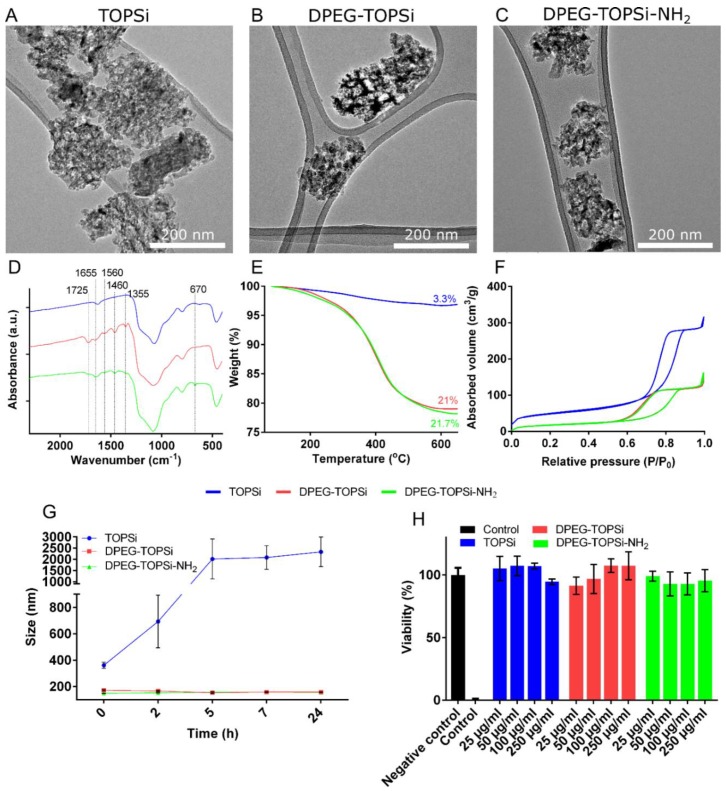
Characterization of the nanoparticles: (**A**–**C**) TEM images of TOPSi, DPEG-TOPSi, and DPEG-TOPSi-NH_2_ NPs, (**D**–**F**) FT-IR spectra, TGA, and N_2_ ad/desorption curves (TOPSi (Blue), DPEG-TOPSi (Red) and DPEG-TOPSi-NH_2_ (Green)), (**G**) Colloidal stability of the nanoparticles in PBS at 37 °C at different time points, and (**H**) Viability of macrophages when incubated with different nanoparticles.

In order to improve the stealth properties of PSi NPs, we decided to employ a dual-PEGylation strategy, aiming to enhance masking of the surface against immunorecognition. Additionally, the dense PEG layer could stabilize the TOPSi particles from enzymatic hydrolysis, which has been previously observed for the thermally oxidized PSi and reported by us and other groups [37,38]. The irregularly elongated shape of the TOPSi nanoparticle was not affected by the surface modifications as observed with TEM (Figure 1A–C). However, after the dual-PEGylation the particles were more separated instead of being as particle clusters because dual-PEGylation improved their dispersion. The success of the chemical modifications were verified with FTIR (Figure 1D). In the spectrum of DPEG-TOPSi, the 1355 cm^−1^ and 1460 cm^−1^ emerges from the –CH_3_ and –CH_2_ bendings, respectively. The peaks at 1560, 1630, 1655, and 1725 cm^−1^ are from amide II bond, –O–H, amide I bond, –C=O stretching, respectively. There is an amide bond in the 2.0 kDa PEG-silane and thus the amides and –C=O group were observed in the spectrum of the DPEG-TOPSi [34]. The peak at 870 cm^−1^ in DPEG-TOPSi-NH_2_ was denoted to be due to the N–H wag indicating the successful surface modification on the DPEG-TOPSi-NH_2_ NPs. The amount of the added organic molecules was studied with TGA (Figure 1E). The weight losses for TOPSi, DPEG-TOPSi, and DPEG-TOPSi-NH_2_ were 3.3 ± 0.2 wt %, 21.0 ± 0.9 wt % and 21.7 ± 1.4 wt %, respectively. Thus, the DPEG and –NH_2_ content were 17.7 wt % and 0.7 wt %, respectively. The pore characteristics were studied with N_2_-sorption (Figure 1F). All the samples displayed the hysteresis effect that is typical for mesoporous materials. The dual-PEGylation decreased the surface area and the pore volume (Table 1) as similarly observed previously by Nissinen et al. (2016) [15]. The enhanced colloidal stability was studied by incubation the NPs in PBS (Figure 1G). The dual-PEGylation improved significantly colloidal stability of the suspension; TOPSi aggregated readily, but the DPEG-TOPSi was stable during the experiment. The amine addition in the sample of DPEG-TOPSi-NH_2_ did not cause significant effect on its colloidal stability within 24 h. Finally, the NP biocompatibility was confirmed after interaction with the RAW macrophages. No significant differences were observed in any of the samples compared to the control (untreated cells).

### 3.2. Radiolabeling of [^111^In]In-DPEG-TOPSi and [^111^In]In-TOPSi

**Scheme 1:**
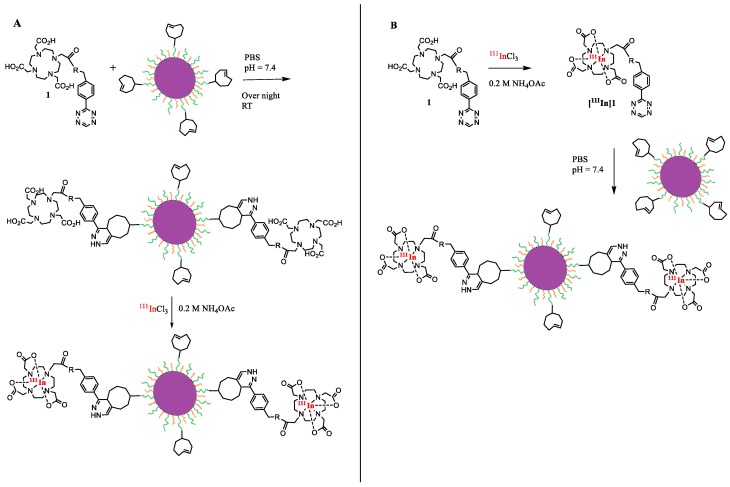
^111^In-radiolabeling of DPEG-TOPSi particles with approaches **A** and **B**.

Previously we have reported biological evaluation of several ^111^In-labeled PSi NPs [39,40]. Regardless of the surface chemistry, the particles have exhibited limited in vivo stability resulting to some released ^111^In species and complicating quantification of the NP biodistribution at later time points. Compromised radiochemical stability is a significant disadvantage especially when considering the use of long circulating NPs, because making difference between the NP-bound and plasma protein bound radiolabel is extremely difficult. Therefore we decided to investigate two different approaches for radiolabeling of the TOPSi NPs, one with a one-step radiolabeling of the NPs in which the NPs were functionalized with the ^111^In chelating DOTA-PEG_4_-Tz (**1**) prior to the labeling (Approach **A**) and another in which the [^111^In]In-DOTA-PEG_4_-Tz ([^111^In]**1**) was first synthesized and then conjugated to the NPs using the inverse electron-demand Diels-Alder click chemistry (Approach **B**) (Scheme 1).

**Scheme 2:**
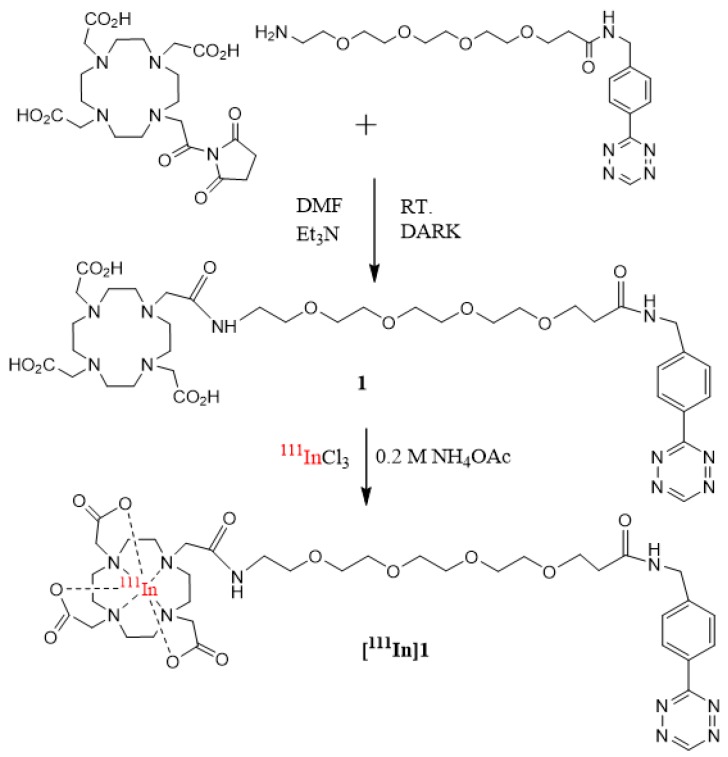
Synthesis and radiolabeling of [^111^In]In-DOTA-PEG_4_-Tz ([^111^In]**1**).

The labelling precursor DOTA-PEG_4_-Tz (**1**) was successfully synthesized through an amide coupling reaction between a DOTA-NHS ester and Tz-PEG_4_-NH_2_ with 78 ± 2% yield (*n* = 3) (Scheme 2 and Figure S4). In approach **A**, BCN- or TCO-functionalized DPEG-TOPSi particles were conjugated first with DOTA-PEG_4_-Tz (**1**) and after removal of the unreacted DOTA-PEG_4_-Tz, [^111^In]InCl_3_ was used for labeling. The one-step ^111^In-labeling approach **A** gave high labeling radiochemical yields (50 ± 11%), regardless of the original BCN or TCO functionalization. In approach **B**, [^111^In]In-DOTA-PEG_4_-Tz ([^111^In]**1**) was first synthesized. Radiochemical yield of [^111^In]**1** was >99% when at least 8 µg (9.75 nmol) of precursor **1** was used (Scheme 2). The synthesized [^111^In]**1** was conjugated to BCN- and TCO-functionalized DPEG-TOPSi NPs through the IEDDA reaction in PBS (pH = 7.4) with radiochemical yields of 13 ± 3% (*n* = 4) and 40 ± 8% (*n* = 4), respectively. Due to the significantly higher radiochemical yields, we decided to continue our experiments with the TCO-functionalized NPs. The control particles, TCO-TOPSi NPs were radiolabeled with approach **B** within a slightly better radiochemical yield 52 ± 7% (*n* = 3). Particle size for the radiolabeled [^111^In]In-DPEG-TOPSi and [^111^In]In-TOPSi particles (approach **B**) were measured and the hydrodynamic diameter were 226 ± 3 nm and 198 ± 4 nm, respectively.

**Figure 2:**
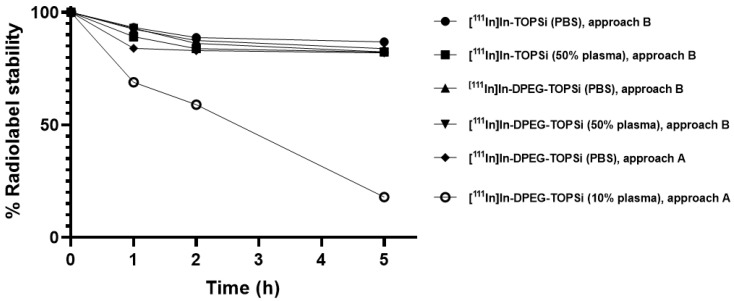
In vitro stability of [^111^In]In-DPEG-TOPSi and [^111^In]In-TOPSi particles in 10% and 50% human plasma and 1 × PBS (*n* = 3). Comparison of radiolabel stability after radiolabeling the NPs with two different methods; either by using the one-step approach **A** in which the NPs were incubated with [^111^In]InCl_3_ or by the two-step approach **B**, in which the TCO-functionalized DPEG-PSi NPs were radiolabeled by using a presynthesized [^111^In]In-DOTA-PEG_4_-Tz ([^111^In]**1**). The radiolabel stability in plasma is decreasing significantly for approach **A** radiolabeled [^111^In]In-DPEG-TOPSi NPs already after 1 h.

Radiochemical stability for radiolabeled particles was determined by incubating the particles in PBS (pH = 7.4) and in 10% and 50% human plasma. The results revealed that those DPEG-TOPSi particles, which were radiolabeled with the one-step approach **A**, exhibited major loss of activity already after 1 h in 10% plasma, while particles radiolabeled with the two-step approach **B**, demonstrated excellent stability in PBS (pH = 7.4) and even at 50% plasma (82% after 5 h in 50% plasma) (Figure 2 and Figure S1). The possible explanation for the observed difference is most probably a weak coordination of the ^111^In^3+^ cation onto the oxidized surface and inside the pores. Obviously, we were not able to completely remove the weak coordination despite of the careful and repeated washings. When exposed to plasma the weakly bound trivalent radiometals can be transchelated with metal complexing proteins such as transferrin [41]. When using the approach **B**, the free ^111^In^3+^ cation is not in a direct contact with the oxidized surface, instead the trivalent radiometal is introduced to the particles complexed with DOTA. The [^111^In]In-DOTA complex has been reported excellent thermodynamic stability for the complex in vivo and supported also by our in vitro findings [42]. Due to their better in vitro stability the NPs radiolabeled by approach **B** were selected for the in vivo evaluation.

### 3.3. Ex Vivo Biodistribution

**Figure 3:**
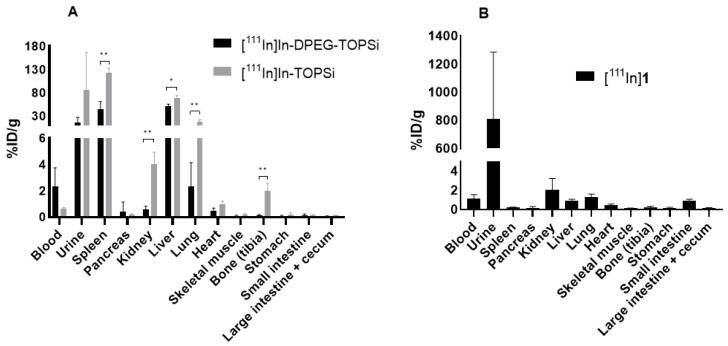
Biodistribution of (**A**) [^111^In]In-DPEG-TOPSi and [^111^In]In-TOPSi and (**B**) [^111^In]In-DOTA-PEG_4_-Tz ([^111^In]**1**) at 1h in Balb/c mice (*n* = 4). Statistical analysis was made by using a *t*-test analysis, significant differences were found in liver, kidney, lung, spleen, and bone (thibia).

Biodistribution of [^111^In]In-DPEG-TOPSi and [^111^In]In-TOPSi particles was evaluated in order to determine how much the dual PEGylation affected blood circulation time of the NPs. Biodistribution of the [^111^In]In-DOTA-PEG_4_-Tz ([^111^In]**1**) labeling agent was also determined as a control. All mice were injected with 100 µg of labeled particles (0.3–0.6 MBq) in 200 µL formulation solution intravenously into tail vein. Blood samples from the contralateral tail vein were taken at the selected time points to follow the particle residence time in circulation. Mice were sacrificed and the selected tissues were collected for gamma measurements. The [^111^In]In-DPEG-TOPSi particles exhibited a typical biodistribution pattern for NPs with high uptakes in the liver and spleen (44 ± 17%ID/g and 51 ± 5%ID/g at 1 h, respectively) (Figure 3A and Table S1). For the control particles, [^111^In]In-TOPSi, spleen and liver uptakes were even higher (123 ± 10%ID/g and 69 ± 5%ID/g at 1h, respectively). In addition, there was significantly higher lung uptake (18 ± 4%ID/g at 1 h) when compared to the dual-PEGylated particles. The lung accumulation is typical for particles with increasing size, but also surface chemistry of nanoparticles may also influence the accumulation. [^111^In]In-DOTA-PEG_4_-Tz ([^111^In]**1**) was fast cleared through urinary system and no visible uptake in other organs was observed (Figure 3B).

**Figure 4:**
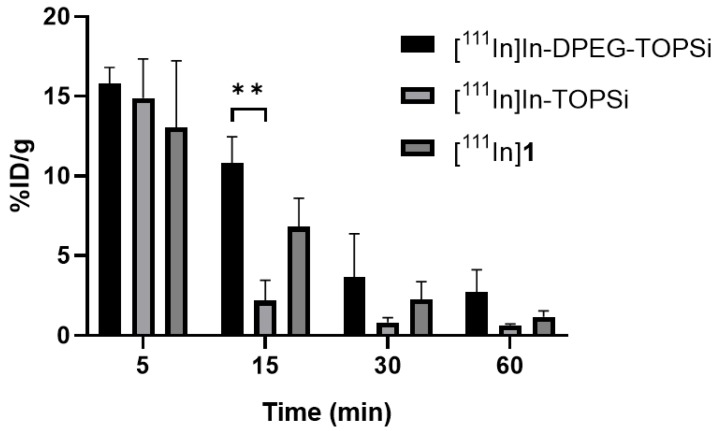
Quantification of resident time in blood for [^111^In]In-DPEG-TOPSi, [^111^In]In-TOPSi and [^111^In]**1** in Balb/c mice (*n* = 4) at 5 min, 15 min 30 min and 1 h after intravenous administration. Unpaired t-test was used to assess the statistical significance of the difference in the of the two particle types in blood, ** *p* > 0.0021.

Blood radioactivity levels for [^111^In]In-DPEG-TOPSi were 15.8 ± 1.0%ID/g and 10.8 ± 1.7%ID/g at 5 min and 15 min after injection (Figure 4). In particular, the value at 15 min is much higher than typically observed for PSi nanoparticles [43,44]. For the control particles, the corresponding values in blood at 5 min and 15 min were 14.9 ± 2.5 and 2.2 ± 1.3%ID/g. [^111^In]In-DOTA-PEG_4_-Tz labelling agent showed still some activity in blood at 15 min (6.8 ± 2%ID/g), but was quickly washed from organs as could be seen at 30 min in liver and spleen (1.4 ± 0.3%ID/g and 0.4 ± 0.3%ID/g, respectively vs. 44 ± 17%ID/g and 51 ± 5%ID/g in liver and spleen for [^111^In]In-DPEG-TOPSi at 1 h). Despite the improved blood residence time of [^111^In]In-DPEG-TOPSi NPs in the circulation, the 4T1 tumor uptake was only 0.2 ± 0.1%ID/g at 1 h and improving only slightly to 0.3 ± 0.2%ID/g at 24 h time point indicating low EPR effect (Figures S2 and S3). This is most probably due the relatively large size of the particles (226 ± 3 nm) compared to what has been reported optimal for the EPR facilitated tumor accumulation [45].

### 3.4. SPECT/CT Imaging

**Figure 5:**
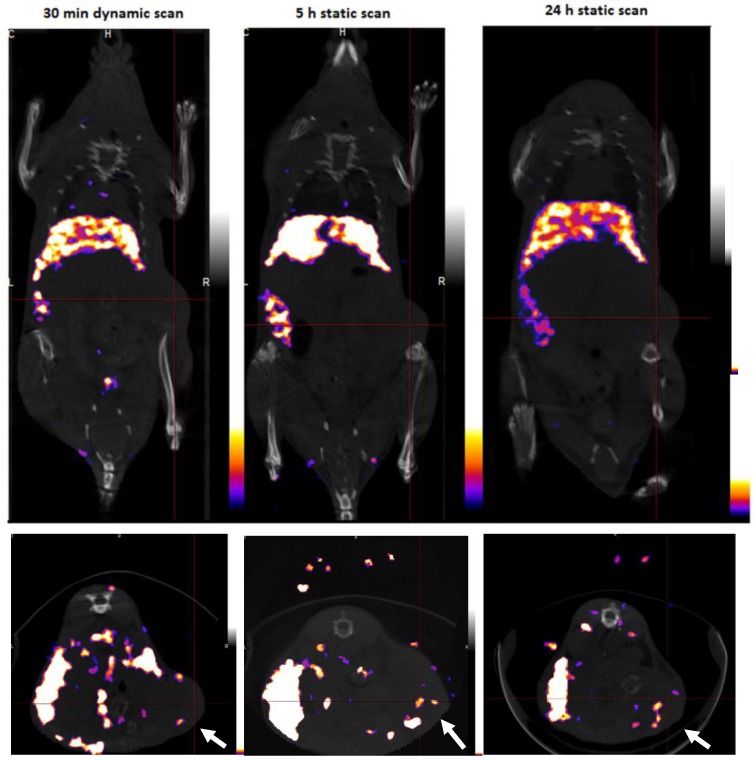
Representative SPECT/CT images (coronal and transversal) from 30 min, 5 h and 24 h time points for [^111^In]In-DPEG-TOPSi particles at 4T1 bearing mice. Location of the tumor is indicated by a cursor. Highest radioactivity levels were observed in liver and spleen.

[^111^In]In-DPEG-TOPSi particles (100 µg, 2–4 MBq) were administered intravenously via the tail vein and the animals were imaged under isoflurane anesthesia at 30 min, 5 h, and 24 h after the NP administration (Figure 5.). Major uptake was seen already after 30 min in the spleen and liver, demonstrating the characteristic accumulation of non-targeted PSi NPs by the mononuclear phagocyte system (MPS). Unfortunately, the tumor activities were too low to be clearly visualized from the SPECT/CT images.

### 3.5. Tumor Autoradiography

**Figure 6:**
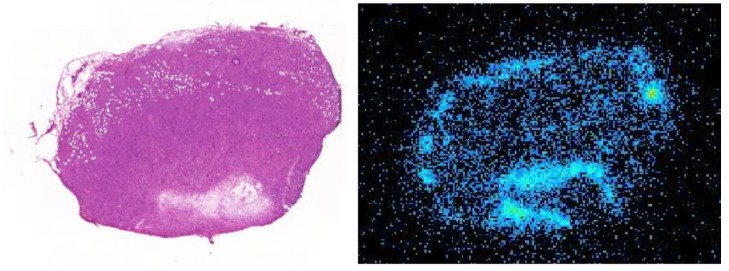
Histological and autoradiography image from 4T1 tumor slice collected at 4 h after [^111^In]In-DPEG-TOPSi administration.

Autoradiographic analysis of the intratumoral radioactivity distribution was carried out for tumors harvested and sectioned at 1 h, 4 h and 24 h after the [^111^In]In-DPEG-TOPSi administration. The tumor sections were imaged by using a BeaQuant real-time autoradiography system. The sections were H&E stained for facilitating the histological orientation of the autoradiography findings. Radioactivity in tumor was heterogeneously distributed showing higher activity levels around the major blood vessels and outer layers possible containing more leaky vasculature (Figure 6 and Figure S5). Less activity was observed in the deeper layers with more solid tumor tissue. Radiolabeled [^111^In]In-DPEG-TOPSi particles (226 ± 3 nm) mainly migrated into the tumor tissue surface but not further to the middle of tumor, which may be due to the relatively large size of the investigated NPs.

## 4. Conclusions

In summary, dual-PEGylated and non-PEGylated TOPSi nanoparticles were successfully radiolabeled with IEDDA ligation by using [^111^In]In-DOTA-PEG_4_-Tz ([^111^In]**1**) as a labeling agent. The two step labeling approach resulted in significantly more stable radiolabeling when compared to a one step approach in which the DOTA conjugated PSi particles were incubated with ^111^In^3+^. The stable radiolabeling allowed us to assess blood circulation time of the radiolabeled particles without interference by the detached and plasma protein bound radionuclide. Biodistribution studies conformed improved blood circulation for the dual-PEGylated [^111^In]In-DPEG-TOPSi NPs compared to the non-PEGylated TOPSi particles. The results demonstrated that the dual-PEGylation efficiently promote the blood circulation time of PSi NPs. However, the longer blood circulation time did not significantly improve the tumor accumulation, most probably due to the suboptimal size distribution of the tested NPs.

## Figures and Tables

**Table 1:** The size, polydispersity index (PDI), zeta potential, and N_2_ adsorption results of the porous silicon nanoparticles.

Measure	TOPSi	TCO-TOPSi	[^111^In]In-TOPSi	DPEG-TOPSi	DPEG-TOPSi-NH_2_	TCO-DPEG-TOPSi	[^111^In]In-DPEG-TOPSi
Size (diameter, nm)	176 ± 6	225 ± 10	198 ± 4	192 ± 1	196 ± 4	276 ± 4	226 ± 3
PDI	0.06 ± 0.03			0.05 ± 0.02	0.04 ± 0.02		
Zeta potential (mV)	−29.3 ± 0.9			−7.2 ± 0.2	12.0 ± 0.8		
BET surface area (m^2^/g)	163 ± 20			60 ± 2	52 ± 9		
BJH volume (m^3^/g)	0.48 ± 0.04			0.26 ± 0.02	0.27 ± 0.03		
BJH pore size (nm)	9.8 ± 0.3			8.8 ± 0.8	10.4 ± 1.5		

## Supplementary Materials

The following are available online at https://www.mdpi.com/1999-4923/11/12/686/s1, Figure S1: In vitro stability of [^111^In]In-DPEG-TOPSi particles in 10% human plasma and 1 × PBS (*n* = 3), Figure S2: %ID/g in tumor for [^111^In]In-DPEG-TOPSi particles at 4T1 tumor model at 5 min, 1 h, 4 h, 24 h and 48 h time points, Figure S3: Tumor/blood ratio and tumor/muscle ratio for [^111^In]In-DPEG-TOPSi particles at 4T1 tumor model at 5 min, 1 h, 4 h, 24 h and 48 h time points, Figure S4: ^1^H-NMR spectra from DOTA-PEG_4_-Tz (**1**), Figure S5: Autoradiography image from 4T1 tumor slices collected at 1 h and 24 h after [^111^In]In-DPEG-TOPSi administration, Table S1: Ex vivo biodistribution of [^111^In]In-DPEG-TOPSi, [^111^In]In-TOPSi and [^111^In]**1** at 1 h time point.

## References

[1] Bae K.H., Chung H.J., Park T.G. (2011). Nanomaterials for cancer therapy and imaging. Mol. Cells.

[2] Wang M., Thanou M. (2010). Targeting nanoparticles to cancer. Pharmacol. Res..

[3] Ehlerding E.B., Chen F., Cai W. (2016). Biodegradable and Renal Clearable Inorganic Nanoparticles. Adv. Sci (Weinh).

[4] Dogra P., Adolphi N.L., Wang Z., Lin Y.-S., Butler K.S., Durfee P.N., Croissant J.G., Noureddine A., Coker E.N., Bearer E.L. (2018). Establishing the effects of mesoporous silica nanoparticle properties on in vivo disposition using imaging-based pharmacokinetics. Nat. Commun..

[5] Chiappini C., Tasciotti E., Fakhoury J.R., Fine D., Pullan L., Wang Y.-C., Fu L., Liu X., Ferrari M. (2010). Tailored Porous Silicon Microparticles: Fabrication and Properties. Chemphyschem.

[6] Tasciotti E., Liu X., Bhavane R., Plant K., Leonard A.D., Price B.K., Cheng M.M.-C., Decuzzi P., Tour J.M., Robertson F. (2008). Mesoporous silicon particles as a multistage delivery system for imaging and therapeutic applications. Nat. Nanotechnol..

[7] Salonen J., Kaukonen A.M., Hirvonen J., Lehto V.-P. (2008). Mesoporous Silicon in Drug Delivery Applications. J. Pharm. Sci..

[8] Shahbazi M.-A., Hamidi M., Mäkilä E.M., Zhang H., Almeida P.V., Kaasalainen M., Salonen J.J., Hirvonen J.T., Santos H.A. (2013). The mechanisms of surface chemistry effects of mesoporous silicon nanoparticles on immunotoxicity and biocompatibility. Biomaterials.

[9] Rosenholm J.M., Mamaeva V., Sahlgren C., Lindén M. (2012). Nanoparticles in targeted cancer therapy: Mesoporous silica nanoparticles entering preclinical development stage. Nanomedicine (Lond.).

[10] Yu M., Zheng J. (2015). Clearance Pathways and Tumor Targeting of Imaging Nanoparticles. ACS Nano.

[11] Godin B., Gu J., Serda R.E., Bhavane R., Tasciotti E., Chiappini C., Liu X., Tanaka T., Decuzzi P., Ferrari M. (2010). Tailoring the degradation kinetics of mesoporous silicon structures through PEGylation. J. Biomed. Mater. Res. A.

[12] Kovalainen M., Kamakura R., Riikonen J., Finnilä M., Nissinen T., Rantanen J., Niemelä M., Perämäki P., Mäkinen M., Herzig K.H. (2018). Biodegradation of inorganic drug delivery systems in subcutaneous conditions. Eur. J. Pharm. Biopharm..

[13] Loh X.J., Lee T.-C., Dou Q., Deen G.R. (2016). Utilising inorganic nanocarriers for gene delivery. Biomater. Sci..

[14] Savage D.J., Liu X., Curley S.A., Ferrari M., Serda R.E. (2013). Porous silicon advances in drug delivery and immunotherapy. Curr. Opin. Pharmacol..

[15] Nissinen T., Näkki S., Laakso H., Kučiauskas D., Kaupinis A., Kettunen M.I., Liimatainen T., Hyvönen M., Valius M., Gröhn O. (2016). Tailored Dual PEGylation of Inorganic Porous Nanocarriers for Extremely Long Blood Circulation in Vivo. ACS Appl. Mater. Interfaces.

[16] Bing X., Wenyi Z., Jisen S., Shou-jun X. (2013). Engineered Stealth Porous Silicon Nanoparticles via Surface Encapsulation of Bovine Serum Albumin for Prolonging Blood Circulation in Vivo. ACS Appl. Mater. Interfaces.

[17] Longmire M., Choyke P.L., Kobayashi H. (2008). Clearance properties of nano-sized particles and molecules as imaging agents: Considerations and caveats. Nanomedicine (Lond.).

[18] Ferreira M.P.A., Ranjan S., Kinnunen S., Correia A., Talman V., Mäkilä E., Barrios-Lopez B., Kemell M., Balasubramanian V., Salonen J. (2017). Drug-Loaded Multifunctional Nanoparticles Targeted to the Endocardial Layer of the Injured Heart Modulate Hypertrophic Signaling. Small.

[19] Gref R., Minamitake Y., Peracchia M., Trubetskoy V., Torchilin V., Langer R. (1994). Biodegradable long-circulating polymeric nanospheres. Science.

[20] Gref R., Lück M., Quellec P., Marchand M., Dellacherie E., Harnisch S., Blunk T., Müller R.H. (2000). ‘Stealth’ corona-core nanoparticles surface modified by polyethylene glycol (PEG): Influences of the corona (PEG chain length and surface density) and of the core composition on phagocytic uptake and plasma protein adsorption. Colloids Surf. B Biointerfaces.

[21] Peer D., Karp J.M., Hong S., Farokhzad O.C., Margalit R., Langer R. (2007). Nanocarriers as an emerging platform for cancer therapy. Nat. Nanotechnol..

[22] Fang J., Nakamura H., Maeda H. (2011). The EPR effect: Unique features of tumor blood vessels for drug delivery, factors involved, and limitations and augmentation of the effect. Adv. Drug Deliv. Rev..

[23] Kobayashi H., Watanabe R., Choyke P.L. (2013). Improving conventional enhanced permeability and retention (EPR) effects; what is the appropriate target?. Theranostics.

[24] Prabhakar U., Maeda H., Jain R.K., Sevick-Muraca E.M., Zamboni W., Farokhzad O.C., Barry S.T., Gabizon A., Grodzinski P., Blakey D.C. (2013). Challenges and Key Considerations of the Enhanced Permeability and Retention Effect for Nanomedicine Drug Delivery in Oncology. Cancer Res..

[25] Moses J.E., Moorhouse A.D. (2007). The growing applications of click chemistry. Chem. Soc. Rev..

[26] Meyer J.-P., Adumeau P., Lewis J.S., Zeglis B.M. (2016). Click Chemistry and Radiochemistry: The First 10 Years. Bioconjugate Chem..

[27] Kolb H.C., Sharpless K.B. (2003). The growing impact of click chemistry on drug discovery. Drug Discov. Today.

[28] Reiner T., Zeglis B.M. (2014). The inverse electron demand Diels–Alder click reaction in radiochemistry. J. Labelled Comp. Radiopharm..

[29] Knall A.-C., Slugovc C. (2013). Inverse electron demand Diels–Alder (iEDDA)-initiated conjugation: A (high) potential click chemistry scheme. Chem. Soc. Rev..

[30] Domingo L.R., Aurell M.J., Pérez P., Contreras R. (2002). Quantitative characterization of the global electrophilicity power of common diene/dienophile pairs in Diels–Alder reactions. Tetrahedron.

[31] Liu F., Liang Y., Houk K.N. (2014). Theoretical Elucidation of the Origins of Substituent and Strain Effects on the Rates of Diels–Alder Reactions of 1,2,4,5-Tetrazines. J. Am. Chem. Soc..

[32] Oliveira B.L., Guo Z., Bernardes G.J.L. (2017). Inverse electron demand Diels–Alder reactions in chemical biology. Chem. Soc. Rev..

[33] Näkki S., Wang J.T.W., Wu J., Fan L., Rantanen J., Nissinen T., Kettunen M.I., Backholm M., Ras R.H.A., Al-Jamal K.T. (2019). Designed inorganic porous nanovector with controlled release and MRI features for safe administration of doxorubicin. Int. J. Pharm..

[34] Näkki S., Rytkönen J., Nissinen T., Florea C., Riikonen J., Ek P., Zhang H., Santos H.A., Närvänen A., Xu W. (2015). Improved stability and biocompatibility of nanostructured silicon drug carrier for intravenous administration. Acta Biomater..

[35] Xu W., Tamarov K., Fan L., Granroth S., Rantanen J., Nissinen T., Peräniemi S., Uski O., Hirvonen M.-R., Lehto V.-P. (2018). Scalable Synthesis of Biodegradable Black Mesoporous Silicon Nanoparticles for Highly Efficient Photothermal Therapy. ACS Appl. Mater. Interfaces.

[36] Donnard J., Berny R., Carduner H., Leray P., Morteau E., Provence M., Servagent N., Thers D. (2009). The micro-pattern gas detector PIM: A multi-modality solution for novel investigations in functional imaging. Nucl. Instrum. Methods Phys. Res. Sect. A Accel. Spectrometers Detect. Assoc. Equip..

[37] Sarparanta M., Mäkilä E., Heikkilä T., Salonen J., Kukk E., Lehto V.-P., Santos H.A., Hirvonen J., Airaksinen A.J. (2011). 18F-Labeled Modified Porous Silicon Particles for Investigation of Drug Delivery Carrier Distribution in Vivo with Positron Emission Tomography. Mol. Pharm..

[38] Alvarez S.D., Li C.-P., Chiang C.E., Schuller I.K., Sailor M.J. (2009). A Label-Free Porous Alumina Interferometric Immunosensor. ACS Nano.

[39] Wang C.-F., Sarparanta M.P., Mäkilä E.M., Hyvönen M.L.K., Laakkonen P.M., Salonen J.J., Hirvonen J.T., Airaksinen A.J., Santos H.A. (2015). Multifunctional porous silicon nanoparticles for cancer theranostics. Biomaterials.

[40] Ferreira M.P.A., Ranjan S., Correia A.M.R., Mäkilä E.M., Kinnunen S.M., Zhang H., Shahbazi M.-A., Almeida P.V., Salonen J.J., Ruskoaho H.J. (2016). In vitro and in vivo assessment of heart-homing porous silicon nanoparticles. Biomaterials.

[41] Ha-Duong N.T., Hémadi M., Chikh Z., Chahine J.M. (2008). Kinetics and thermodynamics of metal-loaded transferrins: Transferrin receptor 1 interactions. Biochem. Soc. Trans..

[42] Mohsin H., Fitzsimmons J., Shelton T., Hoffman T.J., Cutler C.S., Lewis M.R., Athey P.S., Gulyas G., Kiefer G.E., Frank R.K. (2007). Preparation and biological evaluation of 111In-, 177Lu- and 90Y-labeled DOTA analogues conjugated to B72.3. Nucl. Med. Biol..

[43] Rytkönen J., Miettinen R., Kaasalainen M., Lehto V.-P., Salonen J., Närvänen A. (2012). Functionalization of mesoporous silicon nanoparticles for targeting and bioimaging purposes. J. Nanomater..

[44] Sarparanta M., Bimbo L.M., Rytkönen J., Mäkilä E., Laaksonen T.J., Laaksonen P., Nyman M., Salonen J., Linder M.B., Hirvonen J. (2012). Intravenous Delivery of Hydrophobin-Functionalized Porous Silicon Nanoparticles: Stability, Plasma Protein Adsorption and Biodistribution. Mol. Pharm..

[45] Choi H.S., Frangioni J.V. (2010). Nanoparticles for Biomedical Imaging: Fundamentals of Clinical Translation. Mol. Imaging.

