# Supplementary material for: Site-Specific 111In-Radiolabeling of Dual-PEGylated Porous Silicon Nanoparticles and Their In Vivo Evaluation in Murine 4T1 Breast Cancer Model
Source: Pharmaceutics. 2019 Dec 17;11(12):686. doi: 10.3390/pharmaceutics11120686 (PMC6969933; doi:10.3390/pharmaceutics11120686)
Supplement: Supplementary file 1 [file pharmaceutics-11-00686-s001.pdf]

pharmaceutics

MDPI

Supplementary Materials: Site-Specific ^{111}In-Radiolabeling of Dual-PEGylated Porous Silicon Nanoparticles and Their In Vivo Evaluation in Murine 4T1 Breast Cancer Model

Dave Lumen, Simo Näkki, Surachet Imlimthan, Elisavet Lambidis, Mirkka Sarparanta, Wujun Xu, Vesa-Pekka Lehto and Anu J. Airaksinen

Experimental procedures

Materials and chemicals

All chemicals and solvents were obtained from commercial providers and they were used without further purification. Ultrapure water (18.2 MΩ) was prepared on a Milli-Q Integral 10 water purification system. The cell viability assay, culturing media and other procedures for cells used in experiments are described in the *supporting information* (SI). [^{111}In]$InCl_3$ was received from Mallinckrodt Medical B.V. (Petten, The Netherlands).

Cell Culturing. An animal stage IV human breast cancer cell 4T1 was cultured in RPMI-1640 supplemented with 10% FBS, 1% penstrep, 1 x sodium pyruvate, 1 x glutamex in 75 cm^2 flasks incubated at 37 °C in a humidified atmosphere (95%) and 5% CO_2.

Radiochemical stability of the ^{111}In-labeled particles. The stability of the [^{111}In]In-DPEG-TOPSi particles, prepared by both approaches, were investigated *in vitro* in 1 x PBS (pH = 7.4) and human plasma 10%. For the stability tests freshly prepared [^{111}In]In-DPEG-TOPSi particles (0.5 mg) were added to 1 ml of PBS or plasma solution in LowBind Eppendorf tubes and incubated at 37°C under constant shaking. At predetermined time points (1 h, 2 h and 5 h) samples were centrifuged and the radioactivity of pellet and supernatant were measured by dose calibrator (VDC-405, Veenstra Instruments). All assays were carried out in triplicate.

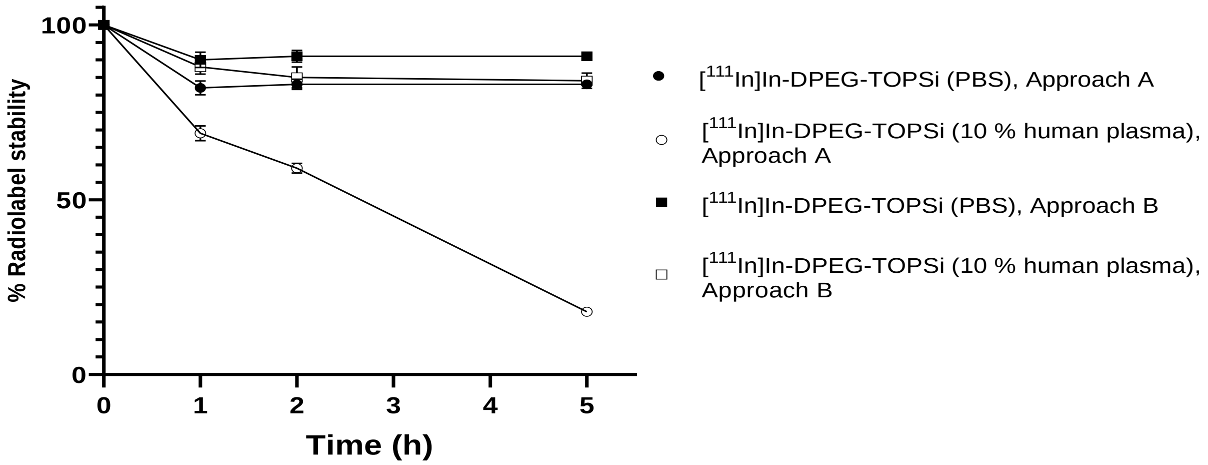

Figure S1. In vitro stability of [^{111}In]In-DPEG-TOPSi particles in 10% human plasma and 1 x PBS (n = 3). The particles were radiolabeled either by using the one step Approach A or the two-step Approach B, in which the particles were radiolabeled by using a presynthesized [^{111}In]In-DOTA-PEG_4-Tz ([^{111}In]**1**).

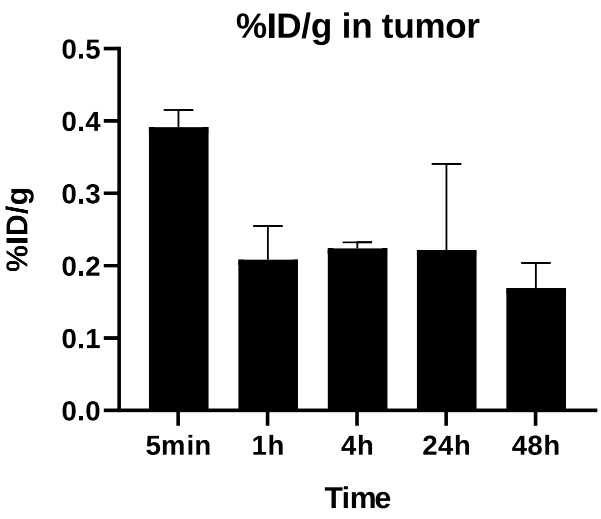

Figure S2. %ID/g in tumor for [^{111}In]In-DPEG-TOPSi particles at 4T1 tumor model at 5 min, 1 h, 4 h, 24 h and 48 h time points.

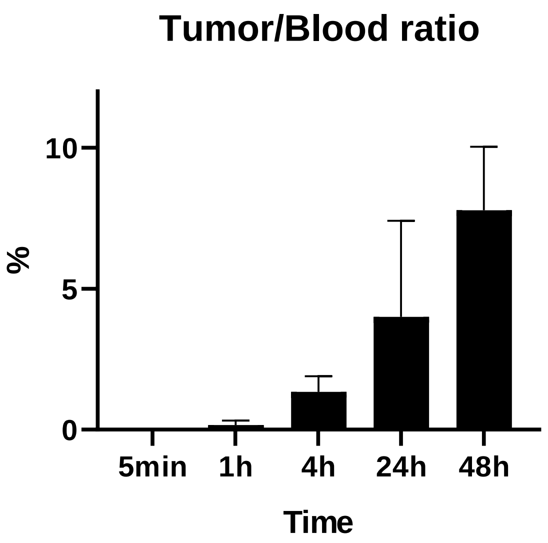

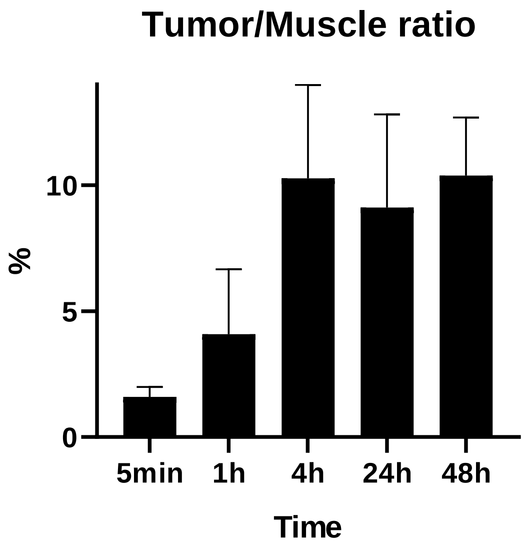

Figure S3. Tumor/blood ratio and tumor/muscle ratio for [111In]In-DPEG-TOPSi particles at 4T1 tumor model at 5 min, 1 h, 4 h, 24 h and 48 h time points.

Table S1. Ex vivo biodistribution of [111In]In-DPEG-TOPSi , [111In]In-TOPSi and [111In]In-DOTA-PEG4-Tz ([111In]**1**) at 1 h time point.

	**[111In]In-DPEG-TOPSi**	**[111In]In-TOPSi**	**[111In]In-DOTA-PEG4-Tz**
Blood	2.32 ± 1.4	0.64 ± 0.08	1.18 ± 0.37
Urine	15.58 ± 11.3	86.23 ± 80.5	810.35 ± 475.2
Spleen	43.72 ± 17.4	122.75 ± 10.4	0.23 ± 0.04
Pancreas	0.44 ± 0.7	0.20 ± 0.04	0.19 ± 0.14
Kidney	0.57 ± 0.3	4.03 ± 0.9	2.03 ± 1.21
Liver	51.38 ± 4.5	69.26 ± 4.6	0.95 ± 0.14
Lung	2.35 ± 1.7	18.04 ± 3.9	1.27 ± 0.34
Heart	0.48 ± 0.2	1.00 ± 0.2	0.47 ± 0.12
Skeletal muscle	0.08 ± 0.06	0.20 ± 0.05	0.14 ± 0.03
Bone (tibia)	0.15 ± 0.05	1.98 ± 0.6	0.26 ± 0.10
Stomach	0.10 ± 0.04	0.21 ± 0.13	0.19 ± 0.06
Small intestine	0.15 ± 0.08	0.15 ± 0.06	0.92 ± 0.17
Large intestine + cecum	0.07 ± 0.03	0.12 ± 0.02	0.16 ± 0.04

^{1}H NMR spectra

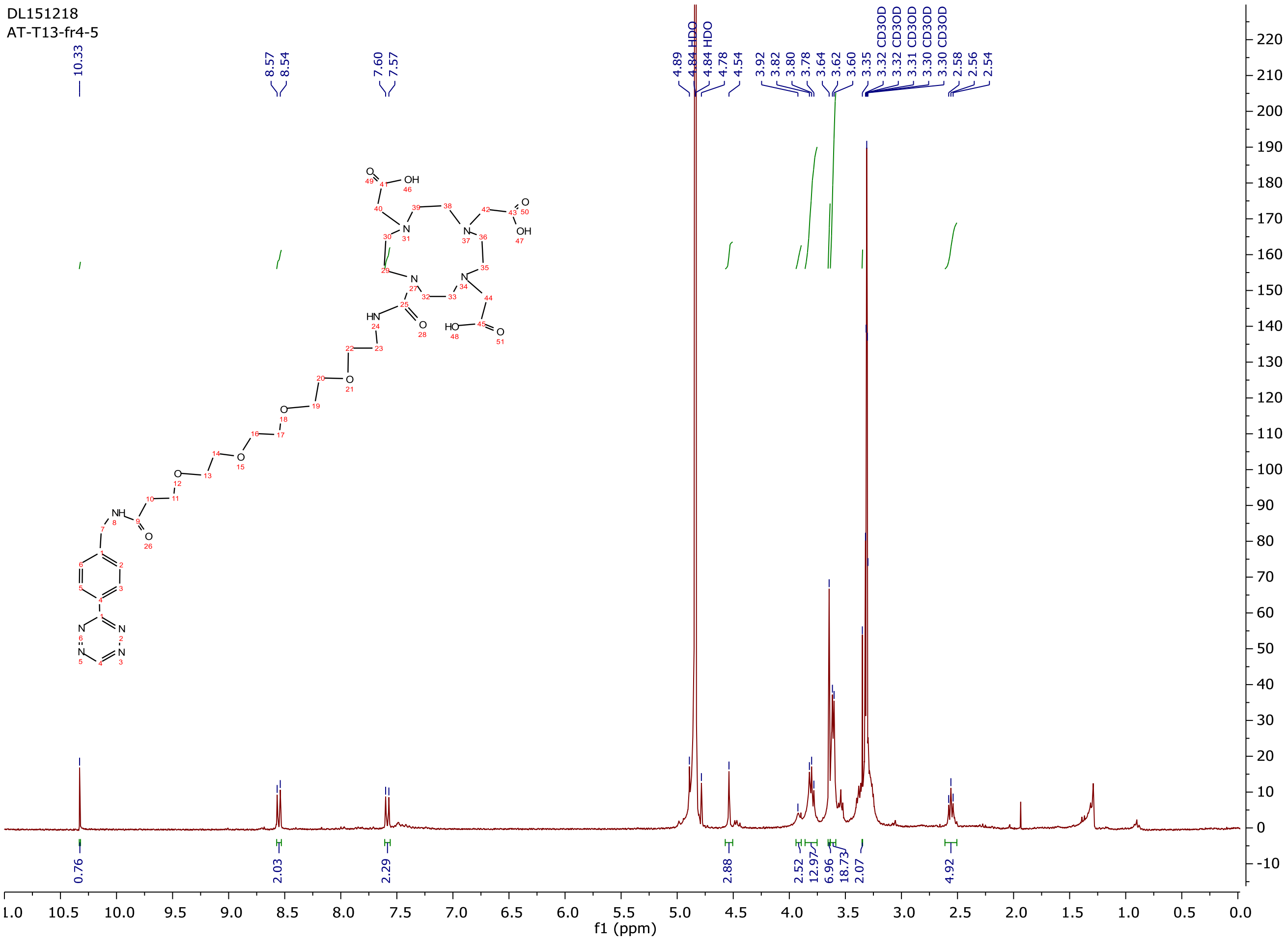

Figure S4. ^{1}H-NMR spectra from DOTA-PEG$_4$-Tz (**1**).

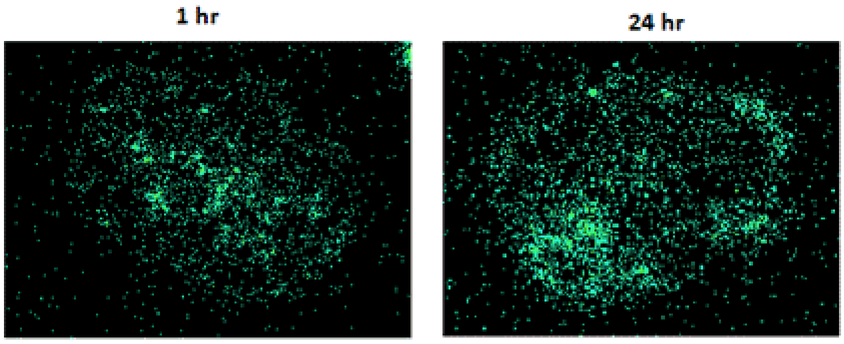

Figure S5. Autoradiography image from 4T1 tumor slices collected at 1h and 24h after [^{111}In]In-DPEG-TOPSi administration.
